# Selecting Mobile Health Technologies for Electronic Health Record Integration: Case Study

**DOI:** 10.2196/23314

**Published:** 2020-10-28

**Authors:** Ryan Shaw, Marissa Stroo, Christopher Fiander, Katlyn McMillan

**Affiliations:** 1 Mobile App Gateway Clinical & Translational Science Institute Duke University Durham, NC United States; 2 School of Nursing Duke University Durham, NC United States; 3 Duke Health Technology Solutions Duke University Health System Durham, NC United States

**Keywords:** mobile health, mHealth, electronic health record, health technology, mobile phone

## Abstract

Mobile health (mHealth) technologies, such as wearable devices and sensors that can be placed in the home, allow for the capture of physiologic, behavioral, and environmental data from patients between clinic visits. The inclusion of these data in the medical record may benefit patients and providers. Most health systems now have electronic health records (EHRs), and the ability to pull and send data to and from mobile devices via smartphones and other methods is increasing; however, many challenges exist in the evaluation and selection of devices to integrate to meet the needs of diverse patients with a range of clinical needs. We present a case report that describes a method that our health system uses, guided by a telehealth model to evaluate the selection of devices for EHR integration.

## Introduction

Mobile health (mHealth) technologies, such as wearable devices and sensors that can be placed in the home, allow for the capture of physiologic, behavioral, and environmental data from patients between clinic visits. This patient-generated health data (PGHD) can help reveal underlying mechanisms of health by filling in gaps in the information, providing insights into the day-to-day activities of an individual, and allowing for better strategies to prevent and manage acute and chronic illnesses. Moreover, with the proliferation of smartphones rising to over 81% of the US population [1] and over 73% of households gaining in-home broadband internet [2], the ability to collect these data from diverse socioeconomic and geographic populations is growing. According to a 2018 survey conducted by Accenture, 75% of US consumers felt that technology was an important part of managing their health [3]. Rapid growth in the global digital health market, estimated to be over US $423 billion by 2024 [4], supports that sentiment. Because mHealth technologies tether to smartphones and Wi-Fi or have cellular-embedded chips, health data can be collected in near real time from patients in their daily environments.

In the United States, over 96% of all nonfederal acute care hospitals now possess a certified electronic health record (EHR) system [5], and over 9 in 10 office-based physician offices have adopted an EHR system [6]. As health care facilities move beyond EHR implementation, the integration of data from connected devices, including mHealth technologies, is gaining speed. Companies such as Apple Inc, for example, have enabled the ability for patients to aggregate their health records on an iPhone from multiple hospitals via authentication by health system patient portals, such as Epic’s MyChart [7]. It is also possible to integrate third-party data, such as patient-generated blood glucose levels, for example, into the EHR system via Apple HealthKit [8]. This capability is possible with many of the major EHR vendors, including Epic, Cerner, and Athena Health, among others. Furthermore, this capability is expanding to Android platforms as well with the use of Google Fit.

While these technologies afford much promise, many challenges exist for health systems and others in the selection of devices to integrate and recommend for equitable patient care. The Office of the National Coordinator for Health Information Technology published a white paper in 2018 highlighting some of the challenges of collecting and using PGHD [9]. These include the technical challenges related to accuracy of measurements, data provenance, and privacy and security concerns. They also explored the patients’ challenges and opportunities, which included the lack of internet or smartphone access as well as health and technology literacy deficits. A 2018 review by Reading and Merrill examined the needs of patients and providers around the use of PGHD in health care. Their review highlighted common needs for technology, including data quality, electronic integration, simple-to-understand actionable insights, and security.

The challenges and opportunities for PGHD are clear, but the path to moving forward remains undefined. One large obstacle is selecting the right devices from the ever-increasing number of consumer digital health devices on the market. Technology selection depends on the data of interest and the technology the patient, clinician, and health care system have ready access to and can use for clinical decision making or population health management. In this use case, we describe a method that Duke University Health System uses to evaluate and select devices for EHR integration.

## Methods

Our team of researchers, clinicians, and informatics technology professionals met to identify factors involved with the selection of devices to integrate with the EHR system. These factors evolve based on feedback from stakeholders and the ever-growing digital health market. Key considerations included clinical validity of devices, patient satisfaction, and usability of both the connected device and the app interface associated with each smart, remote, monitoring device.

**Figure 1:**
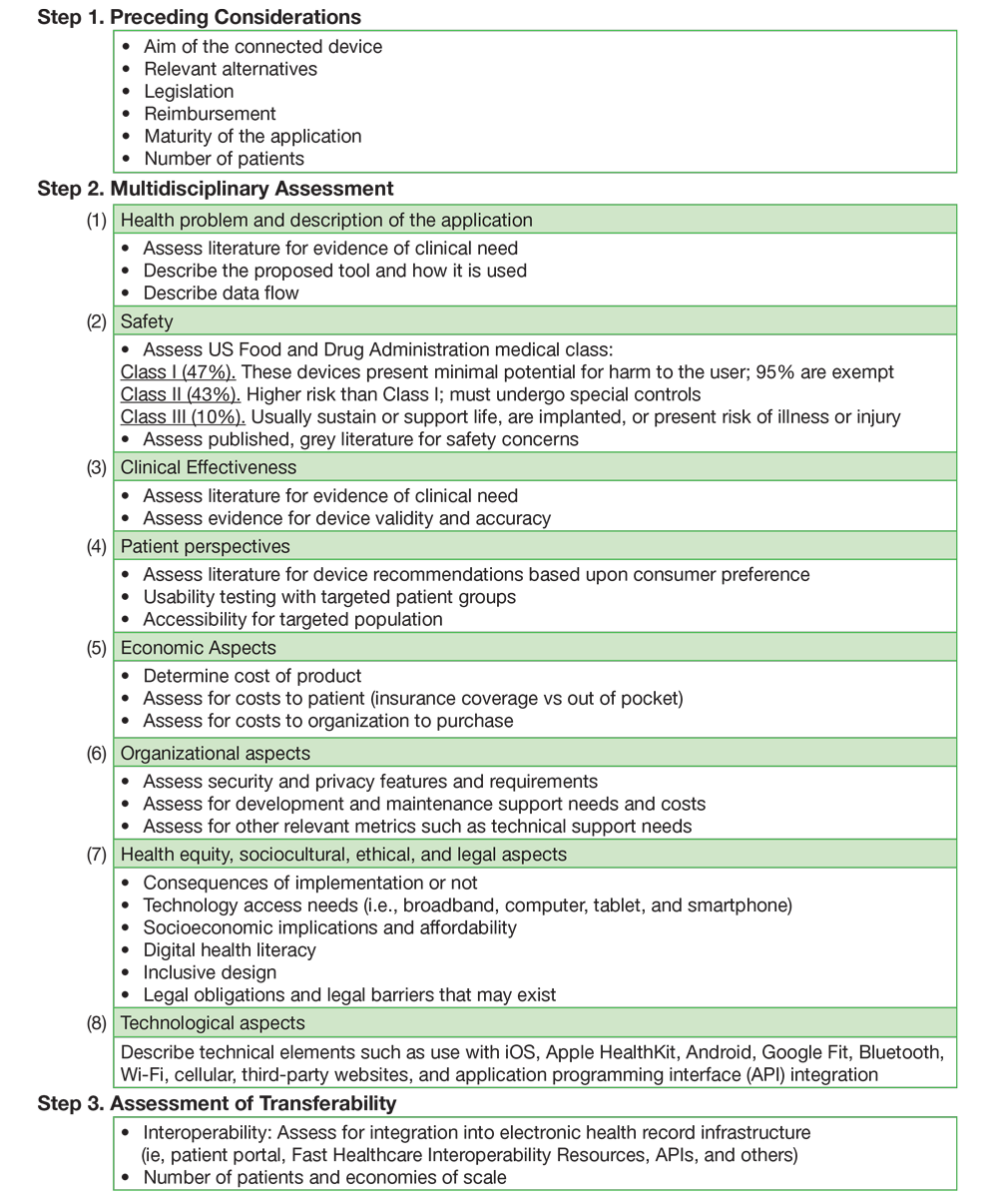
Process for evaluation of connected devices.

We use the Model for ASsessment of Telemedicine applications (MAST) [10] as a guide for device selection. This validated model is used by decision makers to aid in the choosing of the most appropriate telehealth technologies. We have modified the model to reflect variables needed in the selection of mHealth technologies for EHR integration. This includes, for example, details on US Food and Drug Administration (FDA) medical class and technological aspects, such as Bluetooth or Wi-Fi connection. The model includes three steps: Step 1: Preceding Considerations, Step 2: Multidisciplinary Assessment, and Step 3: Assessment of Transferability (see Figure 1) [10].

Step 1 involves determining the purpose of the connected device and relevant alternatives. The goal is to understand the primary outcomes and whether the device involves an upgraded or new technology. Next, several conditions are considered, including the following: legislation (ie, regulations, accreditations, and liability), reimbursement (ie, insurance vs hospital paid), maturity (ie, development time and resources needed over time to support the tool and how safe the tool is), and the number of patients involved to inform an economic analysis. Step 2 then involves a multidisciplinary assessment across eight domains. We added an eighth domain on technological aspects to reflect specific aspects of connected devices. The domains include the following: (1) health problem and description of the application, (2) safety, (3) clinical effectiveness, (4) patient perspectives, (5) economic aspects, (6) organizational aspects, (7) health equity [11], sociocultural, ethical, and legal aspects, and (8) technological aspects. Finally, in Step 3 an assessment is made as to the transferability of connected devices including interoperability (ie, Fast Healthcare Interoperability Resources) and the number of patients who will use the tool to determine costs per patient.

## Results

**Figure 2:**
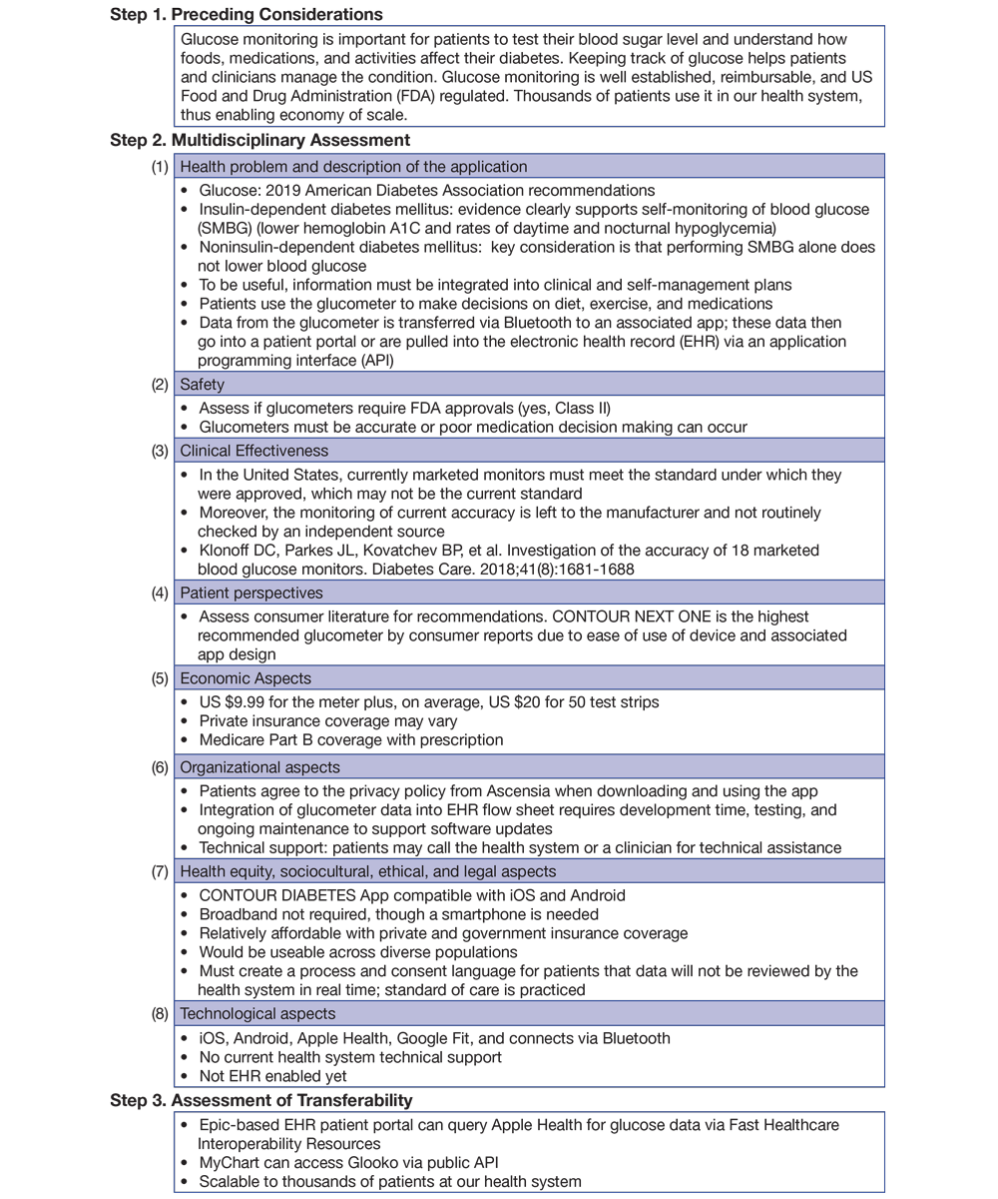
Example evaluation of noncontinuous glucometers: CONTOUR NEXT ONE.

Figure 1 illustrates the three-step process for evaluating and selecting connected devices based upon a modified version of the MAST model. As we evaluate devices, our team maintains an internal working document of a table of devices. This document is refined and expanded as we make decisions and approach device integration. In order to create Figure 1, we began by going through the process of selecting glucometers to recommend be integrated into our Epic-based EHR system (see Figure 2). This exercise allowed us to refine the process and add variables to Figure 1. For example, because the evidence for many devices is limited we expanded to grey literature, including Consumer Reports and Amazon reviews, to gain perspective on patient usability and utility. Other examples include discovering the need to list technical requirements, such as Apple or Android capabilities, connection to Apple Health and Google Fit, and how data are collected and transmitted (ie, Bluetooth, Wi-Fi, and cellular). Of note, this case report focuses on evaluating device selection. Future work will evaluate clinical and institutional outcomes, as these tools are used as part of patient care delivery and research endeavors.

We selected noncontinuous glucometers as our case study (see Figure 2) because of requests from clinician groups to retrieve glucometer data from patients and our experience integrating glucose data into our EHR system via Apple HealthKit [8]. As presented in Figure 2, the exercise revealed that glucose is a data point of value for clinical care. Further, glucometers are considered FDA Class II medical devices and must demonstrate substantial equivalence to a predicate device. A review article by Klonoff et al investigated the accuracy of 18 marketed blood glucose monitors [12]. We searched the consumer-facing literature, such as Consumer Reports, to compare recommendations. The next steps in the evaluation process involved documenting the ability for each glucometer to be used on iOS and/or Android devices, integration with Apple HealthKit and Google Fit, costs, additional technical features, current integration with our EHR infrastructure and how data are retrieved, and if technical support is available. Results showed congruence across these measures and the CONTOUR NEXT ONE glucometer came out as the top contender.

## Discussion

### Principal Findings

The proliferation of wireless and mobile technologies provides opportunities to connect information in real-world settings via wearable sensors and, when coupled with fixed sensors embedded in the environment, to produce continuous streams of data on an individual’s biology, psychology, behavior, and daily environment. These collected data have the potential to be analyzed and used in real time to prompt individuals to change behavior or their environmental exposures that can reduce health risks or to optimize health outcomes.

Selecting devices for integration requires many factors to be evaluated. These factors are technical, clinical, organizational, economic, and patient focused. Popular and currently well-known devices, such as the Fitbit and Apple Watch, are easier to identify due to their accessibility and widespread adoption. Evidence needed for activity trackers like these to be on the consumer market is also less stringent compared to evidence needed for a portable electrocardiogram or glucometer, which require FDA clearance. Devices that require FDA clearance provide an additional layer of evidence for safety and utility. This is in contrast to devices that do not require FDA clearance, such as sleep monitors.

Continued discussion with clinical and operational leaders suggests how broad the idea of technical support could be. Technical support can include such steps as configuring the device for the patient, providing support in person or remotely, and having staff available for ongoing troubleshooting. Other levels of technical support include patient support for managing the clinical data landing in the EHR system, with or without notification, along with addressing support related to the notifications specifically. Lastly, technical support should implicitly include the presentation of the data to providers so that they are actionable and accessible. Actionable and accessible data are essential for the provider or care manager to be able to intervene, while also not exacerbating provider burnout, which is more frequently reported since the large-scale implementation of EHRs. While these concepts are fundamental, they are also often frequent attributes referenced as potential barriers to inclusion by clinical and operational leaders.

A variety of devices should be selected for integration so that access to, and accessibility of, these tools is more equitable across patient populations. Patients have both iOS and Android devices and choosing one platform to focus on limits patient accessibility. Further, for devices that connect via a web portal or consume significant data through video, for example, patients may or may not have in-home broadband internet and are limited to internet access via their phone. This could be a limiting factor for patients based upon their geographic location or socioeconomic status. This is also important because the literature shows that devices are not always designed to be accurate across diverse populations. It was reported that the light sensor in some wearable devices was not usable on patients with darker skin tones due to the color of the optical sensor selected. While this has been addressed by many device manufacturers [13], it is a lesson in the importance of ensuring devices are usable across a variety of patient populations.

Future evaluation will also expand to include software platforms, such as those from Livongo, for example, which incorporate a variety of devices and provide personalized guidance to patients and clinicians with chronic illnesses. A third scenario is also necessary to consider regarding integration: there are applications and devices that offer their own portal for viewing data, but do not offer compatibility to iOS or Android, nor can these devices integrate into an aggregator inherently. Given this scenario, it becomes necessary to evaluate a device’s or platform’s capabilities through application programming interfaces (APIs) so that data can be aggregated productively and used in a clinical environment.

While mHealth technologies, specifically connected devices, hold promise to benefit patient care delivery and patient self-management, many challenges exist with their integration into health care. There is limited regulation, and rigorous scientific evaluation of many devices is lacking. There are many devices on the market, and every device must be tested for data quality, interoperability, and usefulness by patients and clinicians. Further, the rapid evolution of the connected device market requires frequent re-evaluation and system software updates. Finally, use of these tools in formal care delivery models is relatively new and, thus, understanding how to support patients and how to integrate and present the wealth of data from devices into actionable insights for clinical decision making continues to advance.

### Conclusions

We present an example on how we recommend which mHealth devices should be integrated into a health system’s EHR system to collect PGHD. Many factors are involved, and it is important to conduct a thorough assessment to assess for clinical requirements, technical features, and patient-level factors such as usability and costs. Figures 1 and 2 can be used as templates for others to expand upon.

## Abbreviations

API: application programming interface
EHR: electronic health record
FDA: US Food and Drug Administration
MAST: Model for ASsessment of Telemedicine applications
mHealth: mobile health
PGHD: patient-generated health data
